# Acoustic transfer of protein crystals from agarose pedestals to micromeshes for high-throughput screening

**DOI:** 10.1107/S1399004714013728

**Published:** 2015-01-01

**Authors:** Christina M. Cuttitta, Daniel L. Ericson, Alexander Scalia, Christian G. Roessler, Ella Teplitsky, Karan Joshi, Olven Campos, Rakhi Agarwal, Marc Allaire, Allen M. Orville, Robert M. Sweet, Alexei S. Soares

**Affiliations:** a Office of Educational Programs, Brookhaven National Laboratory, Upton, NY 11973-5000, USA; b Center for Developmental Neuroscience and Department of Biology, College of Staten Island, The City University of New York, 2800 Victory Boulevard, Staten Island, NY 10314, USA; c Department of Biomedical Engineering, University at Buffalo, SUNY, 12 Capen Hall, Buffalo, NY 14260, USA; d Department of Biological Sciences, Binghamton University, 4400 Vestal Parkway East, Binghamton, NY 11973-5000, USA; e Photon Sciences Directorate, Brookhaven National Laboratory, Upton, NY 11973-5000, USA; f Department of Biochemistry and Cell Biology, Stony Brook University, Stony Brook, NY 11794-5215, USA; g Department of Electronics and Electrical Communication Engineering, PEC University of Technology, Chandigarh, India; h Department of Biological Science, Florida Atlantic University, 777 Glades Road, Boca Raton, FL 33414, USA; i Biosciences Department, Brookhaven National Laboratory, Upton, NY 11973-5000, USA

**Keywords:** macromolecular crystallography, acoustic droplet ejection, crystal mounting, drug discovery, chemical biology, high-throughput screening

## Abstract

Acoustic droplet ejection (ADE) is an emerging technology with broad applications in serial crystallography such as growing, improving and manipulating protein crystals. One application of this technology is to gently transfer crystals onto MiTeGen micromeshes with minimal solvent. Once mounted on a micromesh, each crystal can be combined with different chemicals such as crystal-improving additives or a fragment library. Acoustic crystal mounting is fast (2.33 transfers s^−1^) and all transfers occur in a sealed environment that is in vapor equilibrium with the mother liquor. Here, a system is presented to retain crystals near the ejection point and away from the inaccessible dead volume at the bottom of the well by placing the crystals on a concave agarose pedestal (CAP) with the same chemical composition as the crystal mother liquor. The bowl-shaped CAP is impenetrable to crystals. Consequently, gravity will gently move the crystals into the optimal location for acoustic ejection. It is demonstrated that an agarose pedestal of this type is compatible with most commercially available crystallization conditions and that protein crystals are readily transferred from the agarose pedestal onto micromeshes with no loss in diffraction quality. It is also shown that crystals can be grown directly on CAPs, which avoids the need to transfer the crystals from the hanging drop to a CAP. This technology has been used to combine thermolysin and lysozyme crystals with an assortment of anomalously scattering heavy atoms. The results point towards a fast nanolitre method for crystal mounting and high-throughput screening.

## Abstract 2

An acoustic high-throughput screening method is described for harvesting protein crystals and combining the protein crystals with chemicals such as a fragment library.

## 1. Introduction   

**Figure 1:**
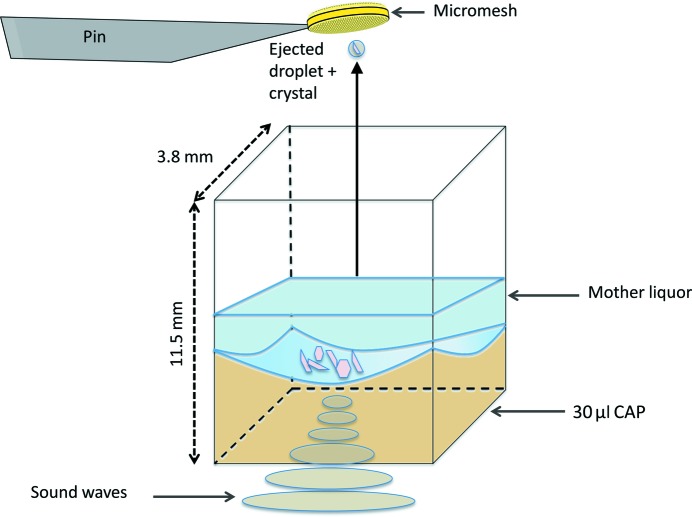
Acoustic droplet ejection (ADE) from a concave agarose pedestal (CAP). ADE uses sound energy to transfer 2.5 nl microdroplets of liquids (such as chemical libraries) or suspended solids (such as mother liquor containing small protein crystals) from a source well, through a short air column (1–10 mm) to a micromesh. Sound energy from the transducer is channeled to the focal point (*i.e.* ejection zone), displacing the surface, where a controlled ejection occurs. The droplet size is governed by the wavelength of the sound emitted; we used a fixed wavelength to eject chemicals and crystals in 2.5 nl increments. Chemicals are ejected from unmodified source wells. Protein crystals are ejected from source wells that have a CAP with the same chemical composition as the mother liquor of the crystals, ensuring that the crystals remain intact and viable for transfer. Agarose, being acoustically transparent, allows the transfer of most suspended solids (such as crystals) with very high precision onto a standard micromesh. Protein crystals in mother liquor are sequestered in the concave basin and suspended above the dead volume. A 2% agarose solution in the random-coil phase (at 100°C) was mixed in a 1:1 ratio with crystallization conditions for lysozyme, thermolysin, stachydrine demethylase and photosystem II. Wells of a 384-well polypropylene source microplate were overfilled with 70 µl of the agarose and precipitant mixture. To create the concave topography of the pedestal, 40 µl were removed from the wells after a 3 s cooling period.

Acoustic droplet ejection (ADE) is an automated and keyboard-driven technology for growing protein crystals (Yin *et al.*, 2014 ▶; Villaseñor *et al.*, 2012 ▶), improving the quality of protein crystals (Villaseñor *et al.*, 2010 ▶) and transferring protein crystals onto data-collection media (Soares *et al.*, 2011 ▶) such as pin-mounted micromesh sample holders. ADE transfers momentum from a sound pulse to move liquids and suspended crystals from the source location through a short air column to an arbitrary destination (Ellson *et al.*, 2003 ▶; Fig. 1 ▶) with a trajectory precision of ∼1.3° for solutions of ∼30 µm crystals.

High-throughput screening of chemical libraries (such as fragment libraries) using X-ray crystallography requires a fast and flexible crystal-mounting technology. Acoustic crystal mounting is an attractive choice for high-throughput screening applications (Table 1 ▶). Since ADE is automated, its success is not dependent on the manual dexterity or physical aptitude of the experimenter. ADE is gentle in that no tools (for example pipette tips) touch the source medium or the destination medium. This prevents contamination, chemical leaching, mechanical stress on crystals or loss of specimen owing to surface adhesion (McDonald *et al.*, 2008 ▶). Transfer is fast (500 transfers s^−1^ for multiple transfers to the same micromesh and 2.33 transfers s^−1^ for transfers to different micromeshes), which simplifies serial applications such as distributing crystals onto different micromeshes and combining each crystal with a different chemical. Acoustically transferring both a crystal and a screened chemical and soaking them together on the same micromesh minimizes the use of protein, chemicals and time.

**Figure 2:**
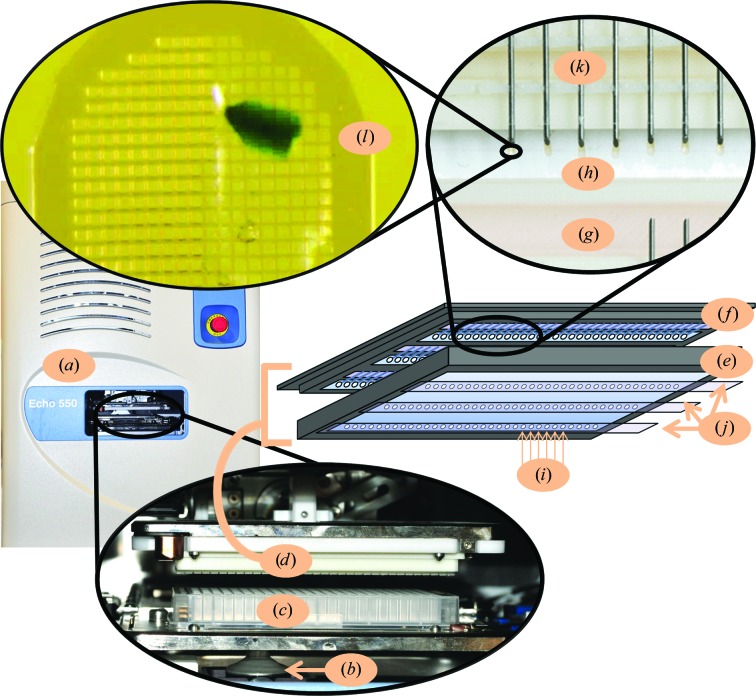
Pin platform box. Crystals and screened chemicals can be transferred in the acoustic transfer chamber of the Echo 550 (*a*) using sound pulses generated by a transducer (*b*) to eject crystals and chemicals contained in a source plate (*c*) into a pin platform box (*d*) (shown without the lid for clarity; Yin *et al.*, 2014 ▶). The pin platform lid (*e*) isolates the pin platform (*f*) to prevent dehydration. The internal environment is governed by mother-liquor solution that is secured in 1% agarose and is deposited into a moat (*g*) in the pin platform. The window (*h*) is used to view specimens and to add components through apertures (*i*) in the lid. After all of the crystals are mounted, tape is used to seal the apertures (*j*). The pin platform box contains 96 sockets for securing pin-mounted micromeshes (*k*). The crystals are transferred onto the pin-mounted micromeshes. Once mounted, the crystals can be combined with cryoprotectants, heavy atoms, crystal-improving additives or with a fragment library; these chemicals are acoustically transferred from the same source plate (*c*) or from a different source plate. The pin platform box is in equilibrium with the mother liquor before, during and after the crystals and chemicals are transferred onto the micromeshes. The inset (*l*) shows a magnified view of a photosystem II crystal that was transferred onto the micromesh, where it was combined with a chemical. All components of the pin platform box are three-dimensionally printed (print files are available on request).

The ejection trajectory is highly accurate, which allows crystals and screening chemicals to be individually passed from wells in a source plate (described in §[Sec sec2]2) through a small (1 mm diameter) aperture and onto a micromesh that is secured in a sealed pin platform box that contains mother liquor (Fig. 2 ▶). At present, pin-mounted micromeshes are manually snapped into the pin platform, where they are secured in a fitting that mechanically compresses the metal pin (all components are printed by a three-dimensional printer and print files are available on request). Each micromesh is then individually targeted by our acoustic system, so that crystals and screening chemicals can be transferred from a source plate and combined on the micromesh. The pin platform box ensures that the micromesh is in vapor equilibrium with the mother liquor before, during and after the transfer of crystals and screening chemicals. This means that each crystal can be soaked with its screening chemical on a micromesh for as long as desired without the crystal dehydrating. It is also possible to co-crystallize proteins and chemical fragments (or other screened chemicals) *in situ* directly on micromeshes using a similar technique (Yin *et al.*, 2014 ▶).

This study uses the Echo 550 liquid-handling instrument (Labcyte Inc., Sunnyvale, California, USA) to transfer suspended crystals and chemicals from source wells containing CAPs onto micromeshes. Innovations in the Echo line of instrumentation have decreased the ‘dead volume’ (an inaccessible region for ejection) at the bottom of each source well to <4 µl (Harris *et al.*, 2008 ▶). However, crystallization experiments tend to yield few crystals, many of which then disappear into this 4 µl region. Consequently, acoustic crystal transfer is only practical if the crystals are suspended at or near the ejection region.

Here, we describe the use of agarose gels to construct concave pedestals that support protein crystals at a suitable location for acoustic ejection; crystals and chemicals are ejected onto each micromesh for high-throughput screening. The concave pedestals consist of acoustically transparent hydrogels (polymerized matrix materials with high water content). The pedestals suspend protein crystals above the dead volume and sequester them precisely at the ejection zone, where the acoustic ejection pulse occurs. Many types of hydrogels are transparent to acoustic energy. We chose agarose to create concave agarose pedestals (CAPs) for this study because agarose is a safe and common laboratory reagent. In contrast, gelatin pedestals require overnight refrigeration and acrylamide pedestals are made with toxic substances.

Crystals can be pipetted onto CAPs for serial transfer onto micromeshes. The pedestal is impermeable to protein crystals but is permeable to mother-liquor chemicals (so the crystals retain the same chemical composition as the mother liquor). Agarose is acoustically transparent, which facilitates easy and rapid serial transfer of crystals from the CAP to micromeshes. In some cases it may be advantageous to serially transfer microcrystals onto micromeshes in this way, both to save time and to minimize the X-ray background contribution from solvent. However, we believe that the largest utility for acoustic crystal mounting will derive from its ability to readily combine just-mounted crystals with chemicals such as heavy atoms, cryoprotectants, additives that improve crystal quality and of course fragment libraries (Table 2 ▶). We have also grown crystals directly on CAPs to avoid manual transfer (http://www.youtube.com/channel/UCtCiMjlzBnq5VYZzrEi3EiQ). When growing crystals directly on CAPs, agarose is a better choice than agar because the impurities in agar cause the matrix to acquire a yellow tint that can make crystals harder to see.

## 2. Methods   

We used a commercially available Echo 550 liquid-handling instrument (Labcyte Inc) to transfer two standard crystal samples (lysozyme and thermolysin), a metalloprotein sample (stachydrine demethylase) and membrane-protein crystals (photosystem II) from a 384-well polypropylene microplate (Labcyte Inc) source plate onto pin-mounted micromeshes that were secured in a pin platform box (Fig. 2 ▶). The temperature inside the acoustic transfer chamber was tightly controlled at 22°C. The crystals used in this experiment were selected to represent a broad range of crystallization conditions and physical properties, such as fragile rod-shaped thermolysin, rigid cuboidal lysozyme and plate-shaped stachydrine demethylase crystals. The concave agarose pedestals (CAPs) contained the same chemical environment as the crystal mother liquor, including cryoprotectants. Cryoprotection of lysozyme and stachydrine demethylase was with mother liquor plus 15% glycerol (10 ml mother liquor plus 1.5 ml glycerol), thermolysin was soaked in mother liquor plus 20% ethylene glycol (10 ml mother liquor plus 2.0 ml ethylene glycol) and photosystem II crystals were stage-soaked to mother liquor plus 30% glycerol (10 ml mother liquor plus 1, 2 and 3 ml increasing concentrations of glycerol)^1^.

To enable the ejection of all crystals, we pre-loaded the source plate with ∼30 µl CAPs (Fig. 1 ▶). Each CAP was composed of 1% agarose and mother liquor containing cryoprotectant. Each type of protein crystal was separately grown on a cover slip in a standard hanging-drop preparation. The crystals were manually pipetted from their hanging drop onto the CAPs. Each pedestal suspended the crystals above the dead volume that is inaccessible for transfer by the Echo 550. Furthermore, the concave shape of the pedestal concentrated the crystals in the ejection zone (the middle of each source well). Crystals were acoustically transferred from the CAP onto a pin-mounted micromesh (Fig. 2 ▶) and cryocooled for X-ray data collection (cryocooling is described in §[Sec sec3.3]3.3). Subsequent to each crystal-ejection event, the concave shape ensured that the remaining crystals descended to the ejection zone. The concentration of crystals on the CAP determines the average number of crystals ejected with each 50 nl drop (approximately five crystals per micromesh for lysozyme and thermolysin and one crystal per every two micromeshes for stachydrine demethylase and photosystem II; see §2.3). We used thermolysin crystals to measure the time needed to harvest our specimens. The crystal-harvesting rate was found by averaging 15 timed trials of 495 crystal transfers to five distinct locations on 99 micromeshes, which required an average of 212.4 s to complete (Table 1 ▶).

Since each micromesh contains one or a few crystals, additional chemicals can be acoustically added to the already mounted crystals on the micromesh. For example, chemicals from a fragment library can be rapidly distributed so that one or a few crystals on each micromesh are soaked with each chemical fragment. This system allows easy and fast exploitation of protein crystals for high-throughput screening (or serial crystallography) applications such as fragment library screening, cryo-condition search, heavy-atom screening, crystal improvement with additives and fast screening for diffraction quality.

### 2.1. Screening of compatible crystallization conditions   

To demonstrate the general applicability of this crystal-mounting method for samples grown using standard crystallization conditions, we surveyed the chemical compatibility of agarose crystal supports with commercial crystallization screens. 15 µl of crystallization conditions from 96 deep-well commercial crystallization plates, JBScreen Cryo HTS L (Jena Biosciences), Additive Screen (Hampton Research), MemGold (Molecular Dimensions) and MCSG-4 (Microlytic), were dispensed into a 384-well Poly Pro source microplate and centrifuged at 1216*g* for 60 s. The volume in each well was measured using the Echo 550 *WellPing* software and adjusted until all wells contained 15 ± 5 µl. The 384-well polypropylene plate was placed into a hot water bath (shallow enough to keep water out of the wells) and maintained at ∼70°C. 20 ml of a 2% solution of agarose in distilled water was prepared in an Erlenmeyer flask and maintained at ∼100°C on a hotplate until the agarose dissolved. The agarose solution was then cooled to 70°C. It is important to maintain the Erlenmeyer flask with the agarose solution at 70°C, because higher temperatures lead to bubbles and melt the pipette tips, while lower temperatures cause the concave basin to cool asymmetrically. 15 µl of the agarose solution were manually dispensed into each well of the heated 384-well polypropylene plate. Any observed bubbles were ruptured using the pipette tip. The 384-well polypropylene plate was removed from the bath and (after cooling) centrifuged (1216*g* for 60 s). Each CAP was examined for air bubbles, firmness (as verified by probing with a toothpick) and visual evidence of precipitation (owing to incompatibility between mother liquor and agarose). We inspected the concave shape of each CAP. Finally, we added 10 µl of water to each CAP and attempted to eject droplets of this water using the Echo 550.

### 2.2. Protein crystallization and plate preparation   

A 2% agarose solution was heated (100°C for ∼10 min) in a water bath until it reached a random-coil state (a polymer conformation where monomer subunits are randomly oriented but are still bound to adjacent subunits). The agarose solution was then cooled to 70°C and mixed in a 1:1 ratio with the following mother-liquor solutions: 0.2 *M* sodium acetate, 8% NaCl for lysozyme, 0.05 *M* NaOH, 15% ammonium sulfate for thermolysin, 10% glycerol, 10% PEG 3350, 25 m*M* hexammine cobalt chloride, 100 m*M* HEPES pH 7.0 for stachydrine demethylase and 40% PEG 5000 for photosystem II.

In order to achieve a concave basin, the wells must be over-filled with tacky agarose (when cooled to ∼70°C, agarose becomes somewhat adhesive) and mother-liquor solution so that the agarose adheres to the walls of the source well, resulting in a bowl-shaped surface when excess agarose is removed from the center of each well. The wells of a 384-well polypropylene source microplate were overfilled with 70 µl of the agarose and mother-liquor mixture using a pipette. After allowing 3 s for the agarose to adhere to the sides of the well, 40 µl were aspirated out of the well from the center. This created a concave basin in the agarose gel (Fig. 1 ▶). A custom-made positioning tool secured the pipette tip in the center of each well to ensure a symmetric bowl shape.

Crystals of lysozyme (50 mg ml^−1^), thermolysin (50 mg ml^−1^) and stachydrine demethylase (20 mg ml^−1^) were grown by standard hanging-drop protocols (4 µl of protein solution combined in a 1:1 ratio with mother-liquor solution over a 500 µl reservoir). The photosystem II crystals were donated. Crystals were manually pipetted from each hanging drop onto the agarose pedestal, where gravity led them to accumulate in the center. The plate was sealed with adhesive plastic. Using the Echo 550, the supernatant above the crystals was removed in 1 µl increments (by serial ejection onto the plastic adhesive that sealed the source plate; no pin platform box was present) until crystals were observed in the ejecta using a light microscope (supernatant removal). A 1 µl volume was chosen because the emergence of crystals from the CAP was observed to be gradual, so the number of crystals lost in the 1 µl supernatant-removal procedure was small compared with the total number in the well. The adhesive plastic was peeled off after the supernatant was removed. 50 nl of crystal suspension was then acoustically transferred from the CAP to each micromesh (Fig. 2 ▶). In cases where the crystal concentration was high (lysozyme and thermolysin), each micromesh contained an average of approximately five crystals. In cases where the crystal concentration was low (stachydrine demethylase and photosystem II), only mother liquor was ejected onto some of the micromeshes. If crystals were not observed on each micromesh (using a Leica microscope) then additional transfers were made.

Each micromesh that contained crystals was cryocooled. When cryocooling many crystals on pin-mounted micromeshes, the entire pin platform was manually dropped into liquid nitrogen (see §[Sec sec3.3]3.3). When cryocooling only a few crystals on pin-mounted micromeshes, each crystal was individually cooled by hand. Diffraction data were collected on beamlines X12C and X29 at the National Synchrotron Light Source (NSLS). Data sets were processed with *HKL*-2000 (Otwinowski & Minor, 2001 ▶) and further processed using *CTRUNCATE* in the *CCP*4*i* suite (Winn *et al.*, 2011 ▶). Structures were obtained by molecular substitution from published models and were refined using *REFMAC* (Winn *et al.*, 2003 ▶) and *ARP*/*wARP* (Perrakis *et al.*, 2001 ▶) (starting models: lysozyme, PDB entry 1lyz; thermolysin, 4tln; stachydrine demethylase, 3vca; photosystem II, 1fe1; Diamond, 1974 ▶; Holmes & Matthews, 1981 ▶; Daughtry *et al.*, 2012 ▶; Zouni *et al.*, 2001 ▶). Each atomic model was further screened for binding to agarose (ZINC database 87496095) using *AutoDock Vina* (Trott & Olson, 2010 ▶), confirming that the tightest predicted binding pose for agarose monomers has zero electron density^2^ (we could not find any electron density for sugar molecules that might have originated from the agarose gel).

### 2.3. Preparing crystals for screening against a heavy-atom library   

Thermolysin and lysozyme crystals were obtained as described in §[Sec sec2.2]2.2. Crystals were manually transferred from the thermolysin and lysozyme hanging drops to a CAP containing thermolysin mother liquor and to a CAP containing lysozyme mother liquor as described in §[Sec sec2.1]2.1. Additionally, eight water-soluble heavy-atom salt solutions (cupric sulfate, iron chloride, nickel sulfate, hexammine cobalt chloride, potassium iodide, sodium iodide, sodium bromide and copper nitrate) and three insoluble suspensions (platinum chloride, nickel chloride and molybdenum chloride) were added to discrete locations on the same polypropylene source plate. Hence, the same source plate contained all of the building blocks for our screening experiment (the protein crystals and the screened chemicals). To assemble the experiments using these building blocks, two pin platform boxes were loaded with pins and mother liquor (thermolysin mother liquor for the thermolysin crystals and lysozyme mother liquor for the lysozyme crystals; Fig. 2 ▶).

### 2.4. Assessing the acoustic transparency of hydrogels   

Agarose is one example of a class of materials termed hydrogels, most or all of which we predicted to be functionally transparent to the types of sound waves (frequencies, waveforms *etc.*) used for acoustic crystal mounting. To determine the acoustic transparency of various hydrogels, three wells of a 384-well polypropylene microplate were prepared with pedestals of gelatin (3% unflavored gelatin; commercial gelatin), agarose (2% agarose; Sigma–Aldrich catalog No. A6877) and acrylamide [16%(*w*/*v*) 29:1 acrylamide; Sigma–Aldrich catalog No. A7802]. For each hydrogel, we used the Echo 550 *WellPing* software to send five acoustic pulses (11.5 MHz) through the material and to listen to the resulting reflected acoustic signal. The five reflected acoustic profiles from each material were then averaged.

## 3. Results   

### 3.1. Agarose pedestals are compatible with most crystallization conditions   

To test the compatibility of agarose pedestals with common crystallization conditions (Table 3 ▶), 20 µl of each crystallization condition was mixed with 20 µl 2% agarose at 70°C and allowed to cool into a gel. Once hardened, each gel was tested for (i) firmness, (ii) acoustic ejection and (iii) the presence of precipitate. 10 µl mother liquor was added to the gel and 2.5 nl were ejected out of each well onto a plastic cover using the Echo 550. Transfer success was observed under a Leica microscope. A high percentage of wells were both firm enough to support a distinct layer of mother liquor and able to eject this mother liquor (Table 3 ▶). Each well was also examined for precipitation using the Leica microscope. Any solution (agarose and mother liquor) that appeared to form a precipitate was recorded (CAPs were examined with a light microscope and any discoloration was noted as a precipitate). In cases where the initial agarose preparation has a precipitate, adjustment of the agarose concentration and/or the precipitant concentrations usually allowed an effective CAP (data not shown). Crystallization cocktails that stubbornly inhibit gel formation {for example, ammonium sulfate ≥ 30%(*w*/*v*) [30%(*w*/*v*) = 40% saturation] or PEG 5000 ≥ 50%(*w*/*v*)} and prevent droplet ejection can be soaked in the mother liquor after the gel has hardened. We therefore believe that this method is generally applicable to most common protein crystallization conditions.

### 3.2. CAPs eliminate dead volume and reduce loss of crystals   

**Figure 3:**
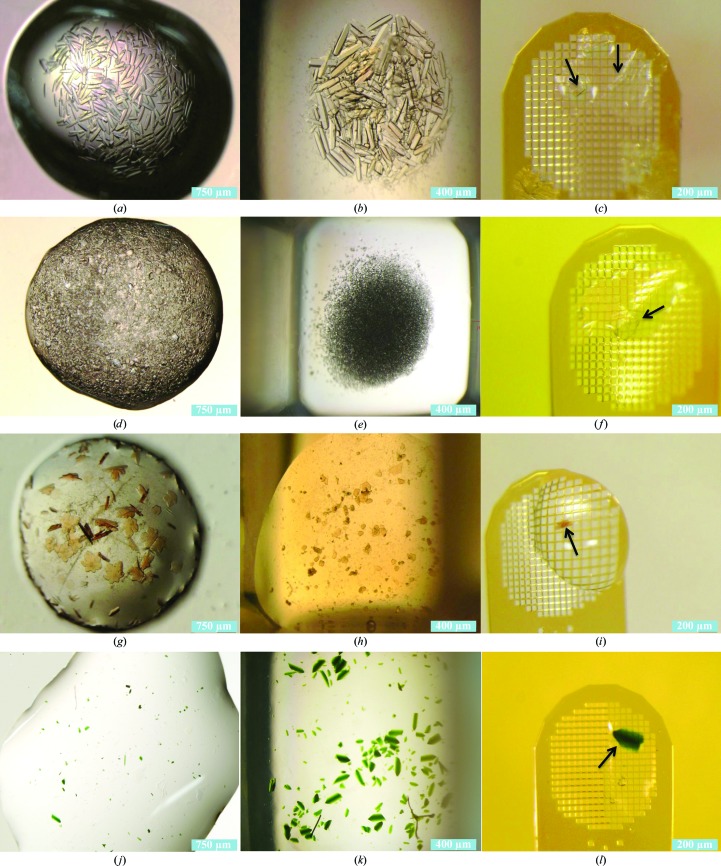
Crystallization, CAP and transfer of thermolysin, lysozyme, stachydrine demethylase and photosystem II. Proteins were crystallized using the hanging-drop method (*a*, *d*, *g*, *j*). Wells were preloaded with a 1% agarose and mother-liquor pedestal. Crystals were transferred manually with a pipette into wells of an acoustically transparent 384-well polypropylene plate (*b*, *e*, *h*, *k*). The concave basin of the CAP caused crystals to concentrate at the ejection zone under the force of gravity. Crystals (indicated by arrows) were transferred onto MiTeGen micromeshes for X-ray diffraction analysis (*c*, *f*, *i*, *l*) (see Supplementary Fig. S2).

Lysozyme, thermolysin, stachydrine demethylase and photosystem II crystals were transferred from their hanging-drop crystallization plates (Figs. 3 ▶ ▶ *a*, 3 ▶ *d*, 3 ▶ *g* and 3 ▶ *j*, respectively) and suspended on CAPs in a source plate. The concave basin assured that many crystals remained in the ejection zone of the wells (Figs. 3 ▶ ▶ *b*, 3 ▶ *e*, 3 ▶ *h* and 3 ▶ *k*). After supernatant removal, the crystals on the CAP were acoustically transferred onto micromeshes (Figs. 3 ▶ ▶ *c*, 3 ▶ *f*, 3 ▶ *i* and 3 ▶ *l*). Diffraction data from acoustically mounted crystals of lysozyme and thermolysin were comparable to diffraction from manually mounted control crystals, demonstrating that acoustic ejection from a concave agarose pedestal does not adversely affect the quality of the data (Table 4 ▶) or of the resulting electron density (see Supplementary Fig. S2). In all cases, the quality of recorded data was compatible with data from manually mounted crystals. *AutoDock Vina* was used to predict the best binding location between each protein structure and agarose monomers; inspection showed that there was no electron density in these areas. Each electron-density map was also visually examined using *Coot* (Emsley & Cowtan, 2004 ▶) to verify that there was no large contiguous difference density that could correspond to a sugar molecule.

### 3.3. Crystals can be combined with chemicals directly on a micromesh   

The thermolysin and lysozyme crystals described in §[Sec sec2.3]2.3 were transferred onto pin-mounted micromeshes that were secured in two pin platform boxes (as described in §[Sec sec3.2]3.2). For each type of protein crystal, 50 nl of crystal suspension were transferred (through apertures) onto each of 36 micromeshes (on average each micromesh contained approximately five crystals). Once all of the crystals were distributed to micromeshes, each heavy-atom solution described in §[Sec sec2.3]2.3 was acoustically transferred (through apertures) onto three different crystal-containing micromeshes of thermolysin and three of lysozyme. Three controls with no heavy atoms were included for each type of protein crystal. Each micromesh with crystals plus heavy atoms was soaked for 1 h. The two pin platform boxes were in equilibrium with the mother liquors of thermolysin and lysozyme (Fig. 2 ▶), so the crystals were soaked without dehydrating. After soaking, the adhesive tape was detached from the back of each pin platform and the lid was removed. Each pin platform (filled with crystal-containing micromeshes) was dropped ‘face down’ into liquid nitrogen, so that the cryocoolant flowed through the window of the pin platform and flash-cooled each of the crystals. Under liquid nitrogen, the pin platform was rotated to face up and each pin-mounted micromesh was manually inserted into a MiTeGen Reusable Base (model B1A-R)^4^.

X-ray data were obtained from all 36 thermolysin heavy-atom soaks and from all 36 lysozyme heavy-atom soaks. The data revealed anomalous signal for some known lysozyme-binding heavy atoms (nickel sulfate and the iodide salts) but not for sodium bromide (which is a lysozyme ligand in a Protein Data Bank structure). Surprisingly, copper sulfate also yielded a detectable anomalous signal when soaked with lysozyme (the PDB did not previously contain a copper derivative of lysozyme). The structure of this derivative was readily solved (PDB entry 4p2e) using the anomalous diffraction from three bound Cu atoms (one at a twofold position near Leu129, another coordinated by His15 and Glu7, and a third discreetly disordered copper near Asp52; similar to Teichberg *et al.*, 1974 ▶). None of the insoluble salts yielded anomalous data when soaked with lysozyme or thermolysin. For both thermolysin and lysozyme, all of the heavy atoms with accessible white lines (excluding iodine and iron) were confirmed to have been transferred by observing a fluorescence peak at the expected energy using a monochromator excitation scan. Table 2 ▶ summarizes the time needed for the Echo 550 to perform soaking experiments of this type.

### 3.4. Hydrogels are acoustically transparent   

**Figure 4:**
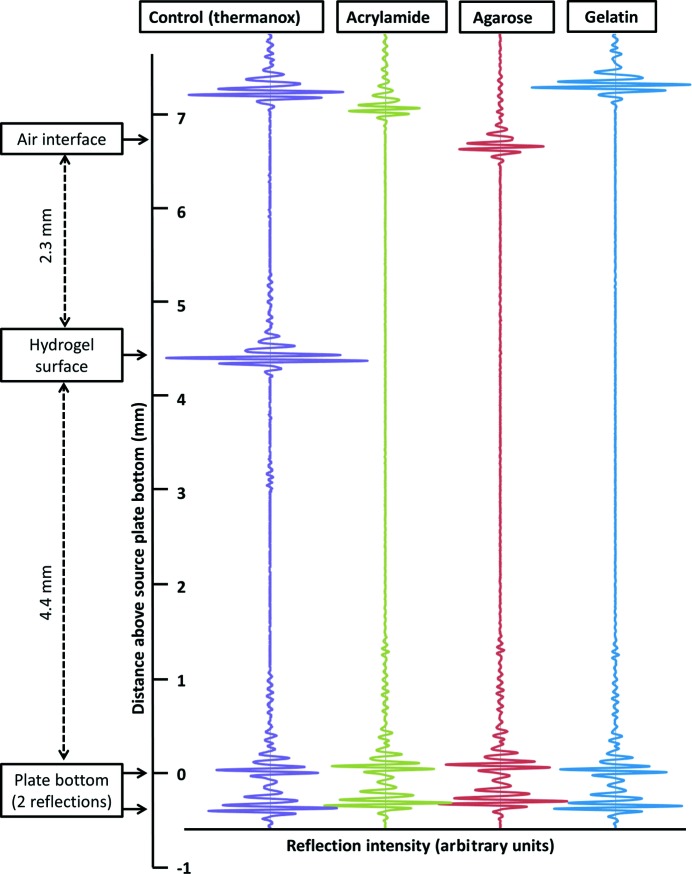
Acoustic transparency of hydrogels. Many hydrogels are acoustically transparent to the waveform and frequency used to transfer crystals (or other materials) onto X-ray data-collection micromeshes (11.5 MHz). The intensity of the reflected sound is shown for hydrogels of acrylamide (green), agarose (red) and gelatin (blue). In all hydrogel cases, a concave pedestal was deposited to a height of 4.4 mm in one well of a 384-well polypropylene plate. Water was then added to a height of 6.7 mm. Five acoustic pings were then transmitted through each well using the Echo 550 and the reflected intensities were recorded as a function of time. The five pulses were averaged for each substance and the averaged values were plotted on a single graph; the horizontal axis is the measured reflected intensity (arbitrary units) and the vertical axis is time. In our control (purple), a Thermanox cover slip was placed on an agarose support to show an example of a material that is acoustically semi-transparent (see Supplementary Fig. S1). Because the speed of sound in all of these substances is virtually identical to that in water, the vertical axis is displayed as a distance (in millimetres). The expected location of the interface between the hydrogel and the water is indicated. Acoustic transfer of crystals from a support matrix to micromeshes can only occur if the largest reflection is from the air–water interface. In the case of the three classes of hydrogels tested, the observed acoustic reflection from the gel–water interface was zero, indicating that all of these materials are possible candidates for positioning specimens at the acoustic focus point.

This study reports the use of agarose gels to support protein crystals at a suitable location for automatic crystal transfer using ADE. Since hydrogels are composed principally of water, we hypothesized that they are likely to be acoustically transparent. Other materials tested for acoustic transparency include gelatin and cross-linked polyacrylamide gels. All of the tested hydrogels were shown to be completely acoustically transparent (Fig. 4 ▶). Cross-linked acrylamide (green) demonstrated no visible reflection at the gel–water interface, but did show noticeable attenuation in the reflected intensity at the water–air interface. This may indicate that some scattering or absorption occurred in the body of the gel, although no reflection was visible at the surface. The scattering/absorption from the 2% agarose gel was much smaller (red), but like the acrylamide there was zero reflection from the gel–water interface. The gelatin (blue) showed no attenuation of the surface reflection and no reflection from the gel–liquid interface. Agarose was selected for this study because it is a common laboratory material, it hardens faster than gelatin and it is safer to work with than acrylamide. However, both gelatin and polyacrylamide were found to be suitable supports for protein crystal transfer using acoustic methods (data not shown). Noncrystallographic applications that could benefit from acoustic touch-less ultralow-volume specimen preparation (such as SAXS and electron microscopy) may be incompatible with agarose supports. In cases where the properties of agarose are found to be unsuitable, other hydrogels may offer an acoustically compatible solution. In cases where the objective is not to eject crystals but rather to monitor crystal growth, an acoustically semi-transparent medium such as Thermanox may be suitable. Recently, 1%(*w*/*v*) agar was used to fabricate a coupling ‘plug’ that conducted sound energy from an acoustic transducer to a crystal suspension at the Linear Coherent Light Source (LCLS; Roessler *et al.*, 2014 ▶). The sound pulses were used to inject crystal containing droplets into the LCLS at a rate of 60 crystal injections per second, matching the LCLS pulse frequency in order to achieve a 60% ‘hit rate’ of X-ray pulses that yielded diffraction patterns.

## 4. Discussion   

Full automation of the high-throughput macromolecular crystal structure determination pipeline would increase productivity in conventional structural biology, as well as enable novel discovery-based solutions to stubborn problems in structural biology (particularly using high-throughput screening of chemical libraries). This goal has been frustrated by the difficulty involved in automating fast transfer of crystals from growth plates onto supports suitable for X-ray data collection. In cases where very high speed is not required, robotic solutions (Viola *et al.*, 2007 ▶), laser tweezer-assisted mounting (Wagner *et al.*, 2013 ▶) and laser-assisted recovery on thin films (Cipriani *et al.*, 2012 ▶) are promising alternatives to manual mounting of individual crystals. For fast serial mounting of crystals of a particular protein, investing time to prepare a CAP allows rapid mounting using acoustic methods. We have demonstrated that acoustic crystal mounting from CAPs will sustain a high rate of 2.33 transfers s^−1^. Combined with automated protein production (Banci *et al.*, 2006 ▶; Gräslund *et al.*, 2008 ▶), crystallization (Bolanos-Garcia & Chayen, 2009 ▶) and end-station automation (Snell *et al.*, 2004 ▶), this will accelerate the output of crystallization facilities to match the data-collection speeds available at next-generation synchrotrons.

Presently, acoustic transfer technology is an advanced method for small-volume liquid transfer. Compared with conventional methods, the acoustic transfer method does not require high-level hand coordination or dexterity. Automated crystal mounting at speeds of several transfers per second prevents loss of crystal viability owing to desiccation, and allows the crystals to be soaked *in crystallo* (on a micromesh) with chemical libraries such as chemical fragments, heavy atoms, cryoprotectants *etc*. Acoustic ejection also eliminates contact between specimens and pins, tips and nozzles, which reduces the risk of cross-contamination with laboratory compounds and contamination by chemicals that leach out of the plastic tubing (McDonald *et al.*, 2008 ▶).

Acoustic micro-mounting with no dead volume (and no lost volume per transfer) is particularly advantageous when purified protein is in limited supply. Advances in protein expression and purification have significantly relaxed the source-material bottleneck in crystallography, but stubborn cases with poorly expressing proteins still occur. Acoustic ejection of protein crystals from CAPs saves scarce purified protein resources by ensuring that all or most of the available protein crystals are rapidly dispensed to micromeshes, where each can be individually combined with chemicals in a high-throughput manner. Acoustic transfer also economizes on chemicals, such as fragment libraries, which are difficult to obtain in large quantities. Thus, acoustic transfer from CAPs allows high-throughput screening of chemical libraries even in cases of crystals of poorly expressing proteins.

Acoustic crystal handling accelerates the rate of specimen preparation to match the rate at which specimens might be examined at modern synchrotron X-ray sources. Automation also has other advantages in addition to speed. A fully automated structure-determination pipeline (including crystal handling) allows a researcher with no laboratory access to orchestrate cutting-edge science by linking the capabilities of automated protein production and purification, automated crystal growth and automated crystal handling and data collection. Full automation will also preserve the intact flow of machine-generated metadata for the full project lifecycle. Most importantly, the automation of specimen handling will make available to all researchers the utility of centrally archived chemical libraries (including fragment libraries, heavy atoms, crystal-improving additives and cryoconditions) because the Echo 550 will be located at a central facility so that chemical acquisition costs can be pooled among a community of users.

Using the strategies outlined here, high-throughput screening can be accomplished rapidly and using limited quantities of protein and chemicals. By sequestering crystals into the ejection zone in a concave basin, most of the crystals in the well can be ejected onto micromeshes. Pre-loaded concave agarose pedestals simplify acoustic crystal transfer and increase yields for easy access to serial crystallography techniques such as ligand screening, cryo-search, heavy-atom screening and crystal improvement.

## Supplementary Material

Supporting Information.. DOI: 10.1107/S1399004714013728/gm5034sup1.pdf

## Figures and Tables

**Table 1:** Characteristics of different crystal-harvesting techniques We used acoustic methods to mount crystals on micromeshes and then to add chemicals that soak into the already mounted crystals [Le Maire *et al.* (2011 ▶) refer to soaking chemicals with crystals that were ready for data collection on plates as *in crystallo* soaking]. Characteristics of robotic crystal-harvesting techniques such as the universal manipulation robot (UMR) are shown in the column headed ‘Robotic’ (Viola *et al.*, 2011 ▶) and manual crystal-harvesting characteristics are shown in the column headed ‘Hand mount’. The time needed to mount crystals was measured for acoustic mounting with the Echo 550 (see [Sec sec2]2). The transfer speeds using other techniques were obtained from published videos (http://www.ruppweb.org/cryscam/umr_small.wmv) or personal communications. The remaining characteristics were obtained from published data (Deller Rupp, 2014 ▶).

	Acoustic	Robotic	Hand mount
Fully automated	Yes	Sometimes	No
Time (s per mount)	0.429 0.0003	120240	>60
Typical mounting media	Micromesh or custom†	Loop or micromesh	Loop or micromesh
Cryoprotectants added to mounted crystal	Yes	Yes	No
Chemical library added to mounted crystal	Yes	No	No
Mechanical stress	None	Small	Operator-dependent
Specific crystal selection	No	Yes	Yes

† Acoustic mounting can deliver specimens to destinations such as direct injection into an X-ray beam (Roessler *et al.*, 2013 ▶, 2014 ▶).

**Table 2:** Time needed for typical serial crystallography applications The transfer rate for the Echo 550 is 500transferss^1^ from a single location and 2.33transferss^1^ when moving between source locations or between destination locations. Approximately 1min is needed to exchange plates. We assume that there are sufficient pin platform boxes pre-loaded with micromeshes for each experiment. The time to complete the two first tasks was measured ([Sec sec3.2]3.2 and [Sec sec3.3]3.3), while for the two last tasks it was simulated using water in place of the chemical library (we have not yet acquired a large chemical library).

Task	Time
Mount crystals onto 96 micromeshes (50nl crystal slurry dispensed to each micromesh, as described in [Sec sec3.2]3.2)	52s
Mount crystals on 36 micromeshes and combine with heavy-atom screen (50nl crystal + 5nl additive, as described in [Sec sec3.3]3.3)	35s
Mount crystals on 96 micromeshes and combine with the commercial Additive Screen kit (50nl crystal + 5nl additive)	1min 32s
Mount crystals on 2000 micromeshes and combine with the fragment library (2000 fragments)	53min 20s

**Table 3:** Crystallization conditions and agarose compatibility screening Four commercially available crystallization plates (each containing 96 different conditions) were screened for incompatibility between the components of the commercial kits and 2%(*w*/*v*) agarose. 20l of each crystallization condition from the commercially available plates were added to the wells of a 384-well polypropylene plate with 20l 2% agarose in the random-coil state. Conditions that resulted in precipitation were recorded. All conditions formed a hardened gel, so this information was not recorded in the table. After cooling, 10l of water were added to the wells and 2.5nl drops of this water were ejected using the Echo 550. Wells from which a drop was not ejected were also recorded.

Commercial crystallization kit	Precipitation	Ejection failure
JBScreen Cryo HTS L (Jena Bioscience)	4 conditions (4.2%)	13 conditions (13.5%)
Additive Screen (Hampton Research)	4 conditions (4.2%)	6 conditions (7%)
MemGold (Molecular Dimensions)	53 conditions (55.2%)	14 conditions (15%)
MCSG-4 (Microlytic)	33 conditions (34.4%)	11 conditions (11%)

**Table 4:** Data-collection and model-refinement statistics Diffraction data from acoustically mounted crystals of lysozyme and thermolysin were comparable to diffraction from a manually mounted control crystal (left columns). Diffraction from acoustically mounted crystals of stachydrine demethylase and photosystem II were typical of these crystals (private communication). In the case of lysozyme and thermolysin, each of the ten data sets from acoustically mounted crystals and each of the ten data sets from hand-mounted crystals was obtained from a single crystal. Where appropriate, average values and standard deviations are shown for each group of ten data sets from similar crystals. In the case of stachydrine demethylase and photosystem II, diffraction data from multiple acoustically mounted crystals were combined into a single data set.

	Lysozyme		Thermolysin		Stachydrine demethylase	Photosystem II
Crystal size (m)	30		50		50	50
Crystallization conditions						
Protein (mgml^1^)	50		50		20	n/a
Buffer	0.2*M* sodium acetate pH 4.6		0.05*M* NaOH		0.1*M* HEPES pH 7, 25m*M* hexammine cobalt	n/a
Precipitant	8% NaCl		15% ammonium sulfate		10% PEG 3350, 10% glycerol	40% PEG 5000
Mounting	Acoustic	Hand	Acoustic	Hand	Acoustic	Acoustic
	Off cap	Control	Off cap	Control	Off cap	Off cap
Data-collection statistics						
No. of data sets	10	10	10	10	1	1
X-ray source	NSLS X12C	NSLS X12C	NSLS X25	NSLS X25	NSLS X12C	NSLS X29
Wavelength ()	0.978	0.978	0.978	0.978	0.978	0.978
Beam width height (m)	150 150	150 150	50 50	50 50	150 150	100 50
Resolution ()	1.89 0.38	2.03 0.36	1.58 0.12	1.66 0.16	3.15	4.9
*R*_sym_ or *R*_merge_ (%)	11.75 3.91	10.94 4.17	8.3 3.2	10.9 4.0	32.7	9.4
Model-refinement statistics						
No. of reflections	12283	11845	44641	39501	9290	32719
Completeness (%)	98.77 2.06	99.14 1.85	99.62 0.58	99.55 0.32	99.60	91.80
*R*_work_ (%)	20.2 0.65	20.2 0.91	14.57 0.32	14.41 0.88	15.00	32.30
*R*_free_ (%)	23.36 0.57	23.29 1.05	17.80 0.50	18.33 1.05	23.60	31.60
R.m.s. deviations						
Bond lengths ()	0.015 0.010	0.014 0.010	0.026 0.003	0.024 0.004	0.010	N/A
Bond angles ()	1.68 0.69	1.60 0.72	2.34 0.31	2.19 0.28	1.470	N/A

## Footnotes

1 Note that the density of the solution to be ejected by the Echo can be larger than expected. For example, adding 1.5 ml glycerol to 10 ml lysozyme mother liquor only increased the total volume by 0.85 ml (43% less than the added glycerol volume). Glycerol also added less than the expected volume to the mother liquors of stachydrine demethylase (30% less) and photosystem II (15% less).

2 For each protein, *AutoDock Vina* was used to identify the tightest binding pose of an agarose sugar dimer to the molecular model. Each model was divided into slabs that were long and wide enough to enclose the entire protein and as thick as our computational memory resources allowed. The photosystem II model was a partial model; consequently, *AutoDock Vina* was only used to search for agarose sugar dimer binding poses in completed areas of the protein

4 We have acquired a Staübli six-axis robot (model TX60) to automate the cryocooling of pin-mounted micromeshes from a pin platform box into a robotic V1 uni-puck. The system was not used for the crystals described here because it was not operational when this work was performed. The design for this system is illustrated in Supplementary Fig. S3. A prototype for the robotic system to transfer pin-mounted micromeshes (with crystals on them) into MiTeGen Reusable Bases and onto a conventional puck lid is shown in Supplementary Fig. S4. .
