# Supplementary material for: Acoustic transfer of protein crystals from agarose pedestals to micromeshes for high-throughput screening
Source: Acta Crystallogr D Biol Crystallogr. 2015 Jan 1;71(Pt 1):94–103. doi: 10.1107/S1399004714013728 (PMC4304690; doi:10.1107/S1399004714013728)
Supplement: Supplementary file 1 [file d-71-00094-sup1.pdf]

BIOLOGICAL
CRYSTALLOGRAPHY

Volume 71 (2015)

Supporting information for article:

Acoustic transfer of protein crystals from agarose pedestals to micromeshes for high-throughput screening

Christina M. Cuttitta, Daniel L. Ericson, Alexander Scalia, Christian G. Roessler, Ella Teplitsky, Karan Joshi, Olven Campos, Rakhi Agarwal, Marc Allaire, Allen M. Orville, Robert M. Sweet and Alexei S. Soares

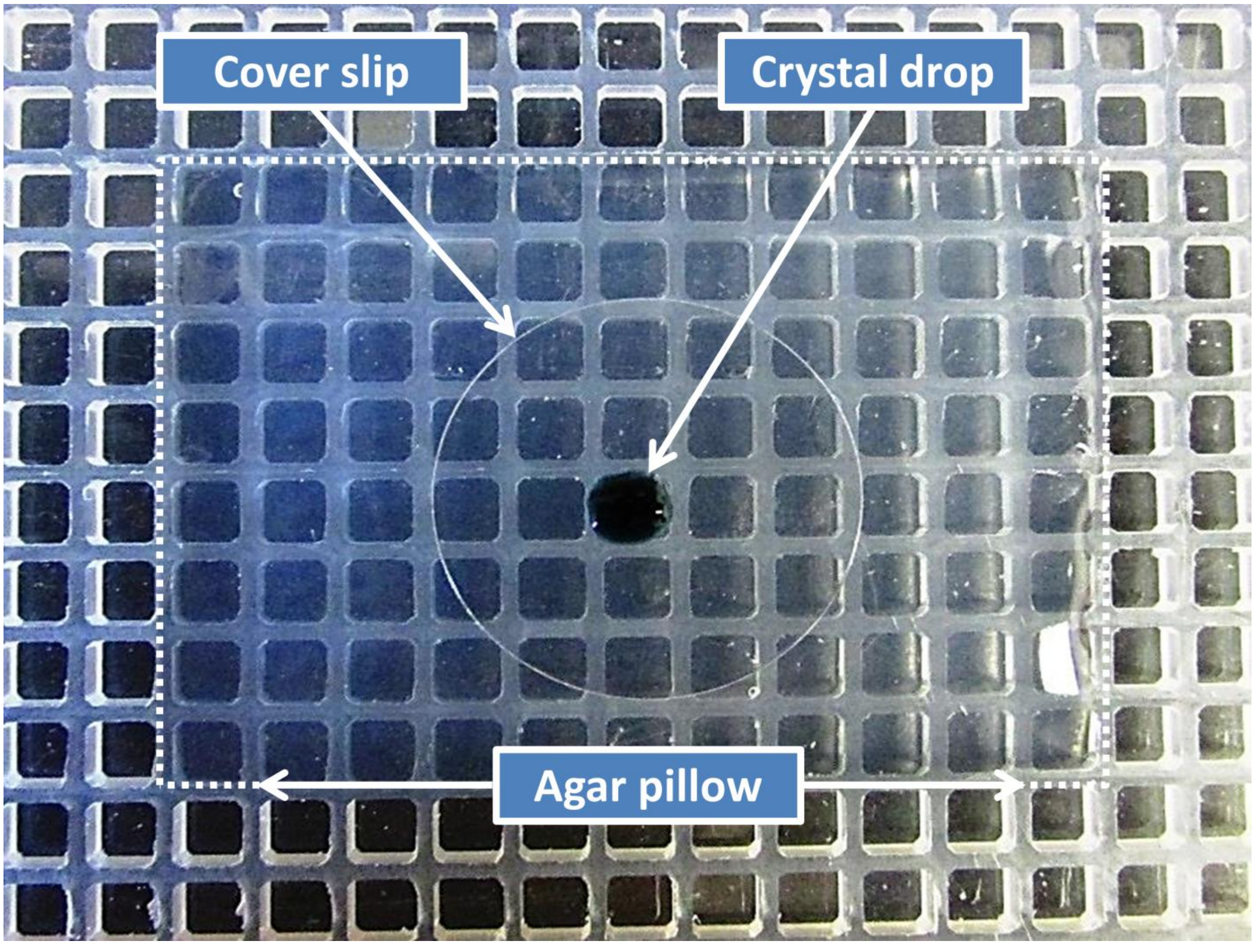

Figure S1 Apparatus for testing acoustic transparency of different materials: We constructed an agar support or “agar pillow” to act as a coupling medium to conduct the sonar sound probe from the Echo 550, through each material being tested, and then through a small drop of water (in this picture, colorant was added to the water for clarity). The agar pillow consisted of 1% agar deposited on a polypropylene acoustic plate that was machined to a total thickness of 5mm high. A commercial polypropylene plate was cut along its flat side until it was half of its normal thickness so that the height of the agar pillow plus the height of the materials being tested would not exceed the maximum sonar probe depth for the Echo 550.

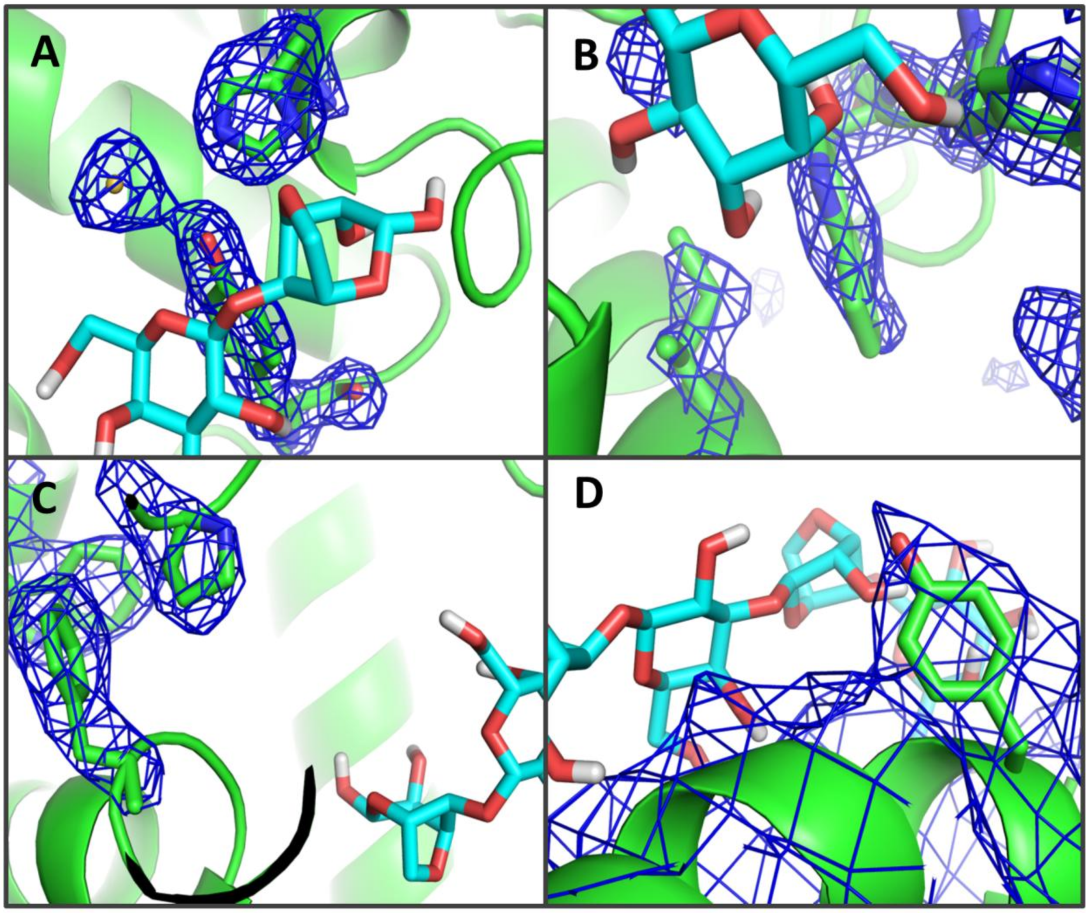

Figure S2 No agar was observed to bind to the crystals of thermolysin, lysozyme, stachydrine demethylase, and photosystem II. (A, B, C, D). Electron density maps and the final refined models are shown for crystals of thermolysin, lysozyme, stachydrine demethylase, and photosystem II. Protein structures were solved using the X-ray data from deposited starting models. Each refined model was screened for binding to agarose monomers using *AutoDock Vina*. Electron density in the vicinity of the tightest predicted agarose binding location is contoured at 2σ for an $F_o\Phi_c$ map ($2F_o$-F_c was not used because we are interested in the *absence* of agarose density). Nearby residues are fully rendered and shown with electron density, the rest of the protein is shown as a cartoon model with no electron density.

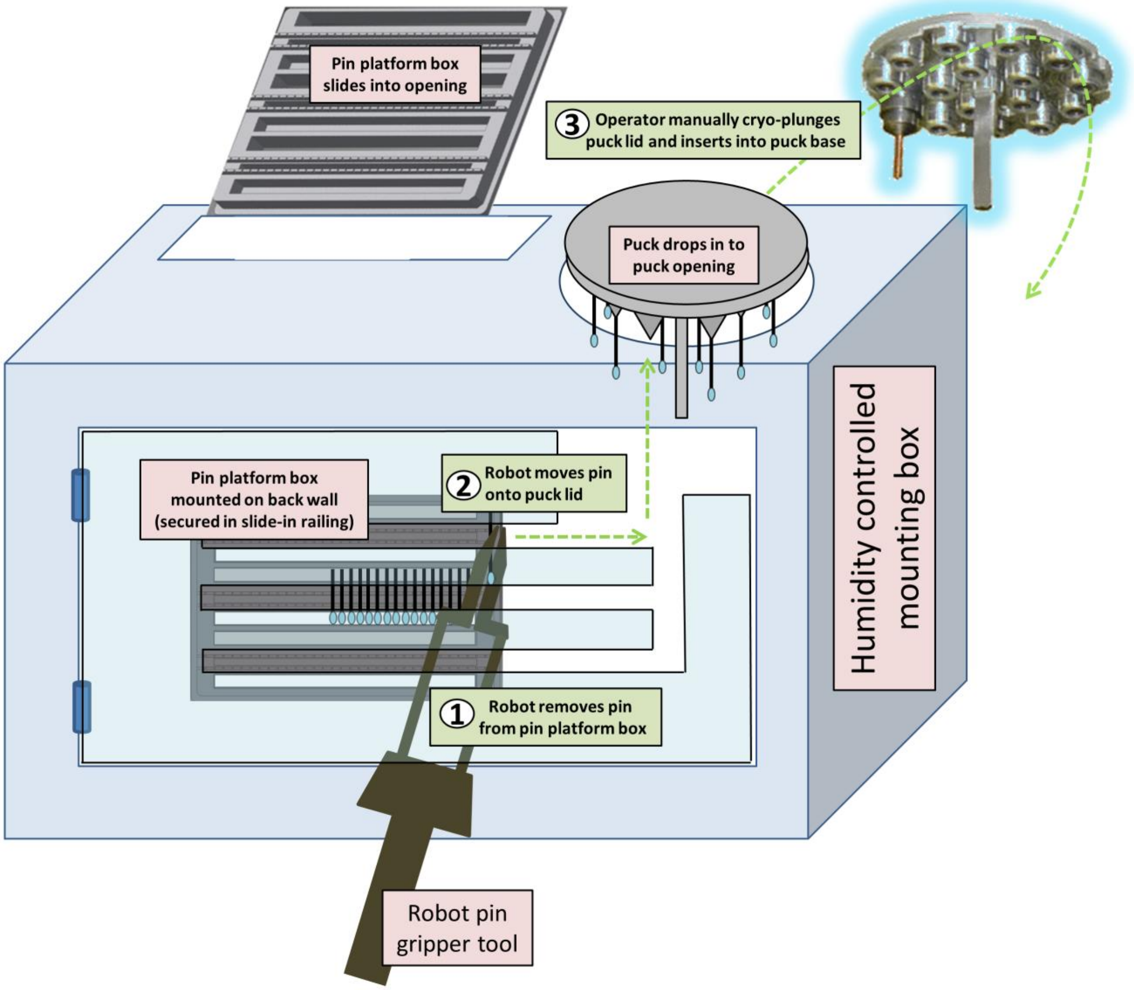

Figure S3 Concept for a robotic system to transfer pin mounted micromeshes (that have been acoustically loaded with crystals) into a conventional robotic puck. We have acquired a Staübli six axis robot (model TX60) to automate the cryo-cooling of pin mounted micromeshes from a pin platform box into a robotic V1 uni-puck. Our robotic pin mounting system was not operational and was not used to mount any of the crystals described in this paper. The humidity in the mounting box is allowed to equilibrate with mother liquor that is contained in a mother liquor tray at the bottom of the mounting box. A pin platform (containing acoustically mounted crystals on micromeshes) is then lowered into the mounting box. A robot controlled pin gripper tool is used to automatically transfer all of the crystal containing specimens from the pin platform and into a robotic V1 uni-puck in three steps:

- Step 1: The pin gripper removes a pin mounted micromesh (containing acoustically mounted crystals) from the pin platform.
- Step 2: The pin gripper inserts the pin into a MiTeGen Reusable Base™ that is magnetically secured onto a V1 uni-puck lid.
- Step 3: After it is filled with specimens, the uni puck lid is manually plunged into liquid nitrogen (using a conventional puck pusher) and inserted into a puck base for data collection.

Supplementary Figure 4 shows a prototype of this robotic pin mounting system.

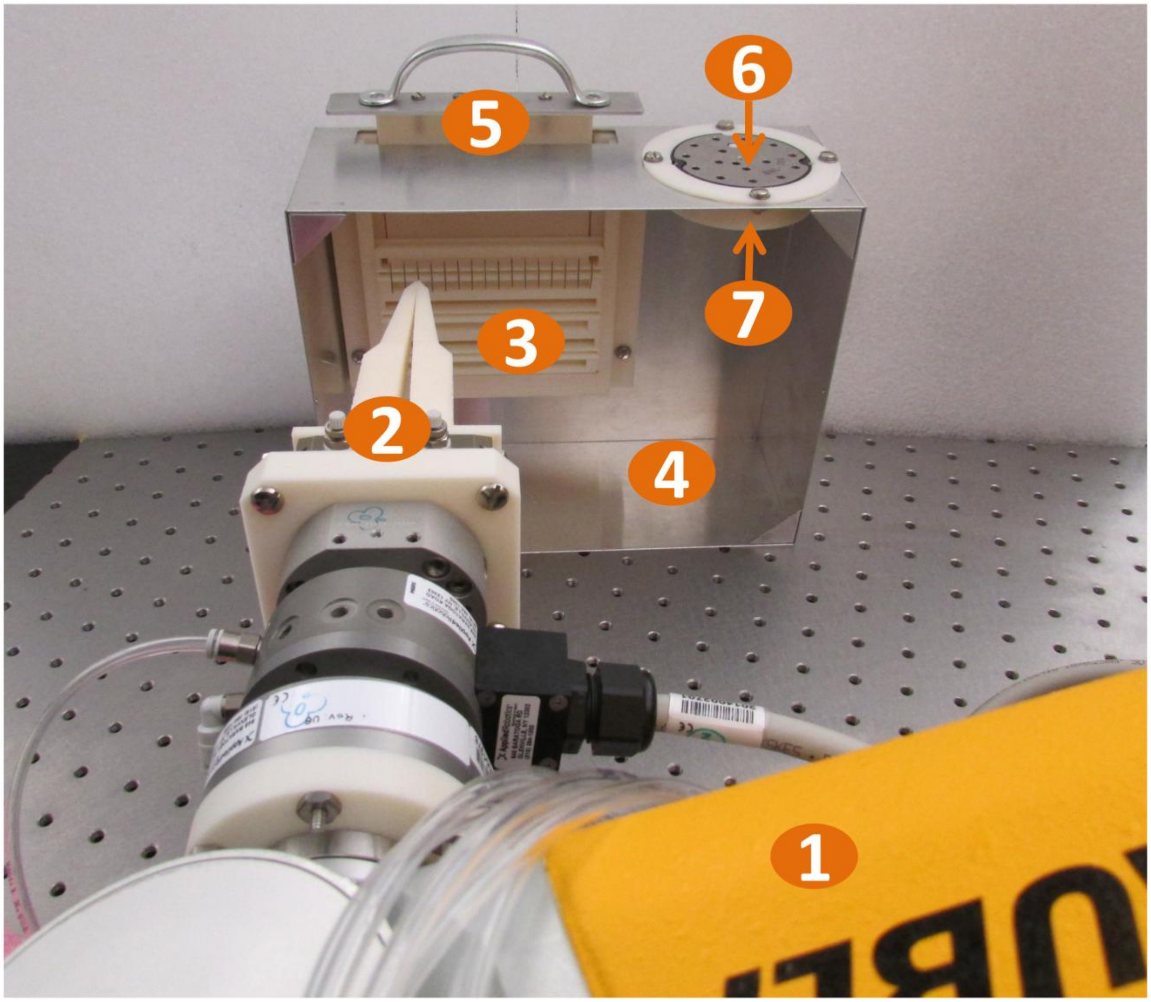

Figure S4 A Staübli six axis TX60 robot (1) is holding a pin gripper tool (2). The pin platform (3), which contains pins (with acoustically mounted crystals) is lowered into the mounting box (4) using a mounting tool (5). The humidity controlled mounting box (shown with no door for clarity) is equilibrated to a mother liquor containing tray (not shown). The pin gripper tool transfers each crystal containing pin to a MiTeGen Reusable Base™ that is magnetically held in a V1 uni-puck lid (6) that slides into a custom puck lid holder (7). When the puck lid is filled, a conventional puck pusher tool is used to manually remove the puck lid from the puck lid holder. The puck lid, containing up to 16 crystal containing micromeshes, is then manually plunged into liquid nitrogen and inserted into a puck base. This prototype was not operational and was not used to mount any of the crystals described in this paper.
